# Phenolic-rich extracts of *Teucrium oliverianum* confer protection against thioacetamide-induced liver fibrosis in rats: Insights from metabolomics, biochemical and histopathological analysis

**DOI:** 10.1371/journal.pone.0330595

**Published:** 2025-09-02

**Authors:** Rania F. Ahmed, Abdelbaset M. Elgamal, Heba A. S. El-Nashar, Noha A. Mowaad, Rania Elgohary, Mohamed A. El-Saied, Mohamed A. Farag, Hiroshi Imagawa, Abdelsamed I. Elshamy, Ahmed M. Abd-ElGawad

**Affiliations:** 1 Department of Natural Compounds Chemistry, National Research Centre, Dokki, Giza, Egypt; 2 Department of Chemistry of Microbial and Natural Products, National Research Centre, Dokki, Giza, Egypt; 3 Department of Pharmacognosy, Faculty of Pharmacy, Ain Shams University, Cairo, Egypt; 4 Narcotics, Ergogenics and Poisons Department, Medical Research and Clinical Studies Institute, National Research Centre, Dokki, Giza, Egypt; 5 Department of Pathology, Faculty of Veterinary Medicine, Cairo University, Giza, Egypt; 6 Pharmacognosy Department, Faculty of Pharmacy, Cairo University, Cairo, Egypt; 7 Faculty of Pharmaceutical Sciences, Tokushima Bunri University, Yamashiro-cho, Tokushima, Japan; 8 Plant Production Department, College of Food & Agriculture Sciences, King Saud University, Riyadh, Saudi Arabia; Yantai Institute of Technology, CHINA

## Abstract

**Background:**

Hepatic fibrosis unfolds as a pathological buildup of extracellular matrix triggered by liver injury. Thioacetamide (TAA) plays a versatile role across various fields—from industrial processes and laboratory research to chemical stabilization. *Teucrium* plants, widely traditional plants, owing to its myriads of pharmacological activities.

**Methods and findings:**

*T. oliverianum* ethanolic (TO-EtOH) and ethyl acetate (TO-EtOAc) extracts were explored for their bioactive metabolites via UHPLC-ESI-qTOF-MS/MS that yielded 48 compounds, mainly flavonoids and phenylethanoid glycosides, alongside phenolic acids, iridoid glycosides, and limonoids. Both extracts showed notable hepatoprotective effects in a thioacetamide (TAA)-induced liver injury model, supporting their therapeutic potential. The TAA group showed a significant increase in AST, ALT, ALP, MDA and TNF-α levels concurrent with a significant decrease of GSH level versus normal control group. In contrast, TO-EtOAC and TO-EtOH administered rats showed a decrease in liver enzymes, including ALT, AST, ALP, total bilirubin, and MDA, and an increase in GSH as compared to the TAA model group. Furthermore, both extracts considerably decreased the overall liver TNF-α content inferring anti-inflammatory action. The histo- and immunohistochemical assays of liver tissue of rats in TAA revealed prominent pathological alterations with bridging fibroplasia in multiple hepatic lobules. A restorative effect that improved hepatic morphology with apparent normal hepatic cells and nominal fibroplasia was evident in the administration of both extracts. Among both extracts, TO-EtOH appeared more effective than TO-EtOAC as manifested by a significant improvement in liver’s biochemical parameters and structural organization.

**Conclusion:**

This study provides robust evidence supporting the antifibrotic effects of *T. oliverianum* in a TAA-induced liver injury model. The anti-proliferative activity and hepatoprotective effects are likely to be mediated by its richness in phenolic acids, flavonoids and phenylethanoids.

## 1. Introduction

Medicinal plants are the most widely used source of natural therapeutics worldwide, attributed to their diverse bioactivities, low cytotoxicity, cost-effectiveness, and superior safety profiles relative to synthetic drugs [1,2]. Due to the presence of bioactive secondary metabolites, medicinal plants and their by-products are increasingly reported as main source of pharmaceuticals [3,4]. *Teucrium* is one of the genera of the Lamiaceae family having over 340 species. The *Teucrium* plants have a long history of use in traditional medicine worldwide for various illnesses, including inflammations, stomach-aches, liver problems, jaundice, abdominal ailments, high blood cholesterol, and digestive issues [5,6]. A review revealed that the plants of this genus possess a variety of biological functions, such as cytotoxic, antispasmodic, antifungal, antibacterial, insect repellent, anti-inflammatory, anti-ulcer, and antioxidant [5,6]. *T. oliverianum* Ging. ex Benth., locally in Saudi Arabia referred to as Qassapa, has been used traditionally to treat a variety of illnesses, especially diabetes [5–7]. Phytochemical screening studies revealed that *T. oliverianum* extracts are rich in several metabolite classes including phenolics, flavonoids, tannins, coumarins, saponins, sterols, terpenes and cardiac glycosides [7]. Phytochemical characterization of this plant led to the isolation and identification of flavonoids [8,9], diterpenoids [7,9,10], iridoids [8,9] and steroids [8,9]. Numerous *in vivo* and *in vitro* assays confirmed efficacy of extracts and metabolites derived from *T. oliverianum* such as anti-nociceptive [11], anti-inflammation [11], insecticide [7], anticancer [8], anti-hypercholesterolemic, and anti-diabetic [10].

Liver cirrhosis is a major global health issue [12], with modern medicine-based therapies often inefficient and expensive [13]. Developing affordable, herbal medicines for treating liver diseases is crucial [3], as evidence shows a strong correlation between medicinal herb bioactive metabolites and liver-protective abilities [1]. Thioacetamide (TAA), a synthetic sulfur compound, is a potent hepatotoxic agent, causing liver injury and dysfunction through reactive oxygen species generation and oxidative stress. [14,15]. Its thiono-sulfur moiety induces liver toxicity through oxidation [16], leading to alterations in hepatocyte membrane permeability [17]. and mitochondrial activity [18], increased intracellular Ca^++^ [19], ultimately causing extensive hepatocellular necrosis [20]. TAA bioactivates to form thioacetamide S-oxide and sulfene, causing hepatic oxidative stress and cirrhosis. This selective hepatotoxicity is used as a rodent model for studying liver fibrosis, cirrhosis, necrosis, and apoptosis [21], leading to chronic inflammation and increased mortality [22].

Accordingly, the primary objectives of the present study were threefold: (i) to comprehensively characterize, using ultra-high-performance liquid chromatography coupled with electrospray ionization quadrupole time-of-flight tandem mass spectrometry (UHPLC-ESI-qTOF-MS/MS), the phytochemical profiles of two distinct extracts derived from the above-ground parts of *T. oliverianum*—specifically, a hydroethanolic extract (ethanol:H_2_O, 7:3; TO-EtOH) and a 100% ethyl acetate extract (TO-EtOAC); (ii) to evaluate the hepatoprotective potential of these extracts in a rat model of TAA-induced liver injury and fibrosis, thereby investigating their efficacy in mitigating liver damage; and (iii) to explore the underlying mechanisms of action through an integrative approach involving biochemical, histopathological, and immunohistochemical analyses.

## 2. Materials and methods

### 2.1 Plant material collection, identification, and authentication

The above-ground parts of *T. oliverianum* were collected from wadi habitat in Qareenah, Riyadh Region, Saudi Arabia (25°03’44.1“N 46°12’10.8”E). The plant collection was carried out during the flowering season (May 2021) in the early morning at 5–7 AM. The plant material was first cleaned in the lab, allowed to air dry in a shaded area, and then crushed into a powder using a grinder (IKA® MF 10 Basic Microfine Grinder Drive, Breisgau, Germany). Prof. Ahmed Abd-ElGawad in Plant Production Department, College of Food & Agriculture Sciences, King Saud University, Saudi Arabia, who specializes in plant taxonomy and ecology, carried out the plant collection, identification and authentication, as reported by [23]. A voucher sample was deposited in the Saudi Arabian National Herbarium and GenBank in Riyadh, Saudi Arabia (TO-4271).

### 2.2 Extraction procedure

The air-dried plant material weighing 900 g was divided into two equal halves, weighing 450 g each. The first sample of the plant material was extracted using ethanol-H_2_O (7:3, 2.5 L) and allowed to stand at room temperature for 5 days before being filtered and this was repeated for 3 times. After collection of ethanolic extracts, solvent was removed using rotavapor at 45 °C until a dark black gum (TO-EtOH; 19.4 g) was obtained. Using the same extraction method, ethyl acetate (100%; 2.5 L) was used to extract the second amount of plant material, and to yield a dark black gum (TO-EtOAC; 17.8 g) upon drying. Both extracts were kept at 4 °C in a fridge until further analysis.

### 2.3 Chemicals and drugs

All solvents were of analytical grade including formic acid (≥95.0%, FA), acetonitrile, water, and methanol (LC-MS grade) purchased from Merck (Darmstadt, Germany). Thioacetamide (TAA, 98%) was purchased from Sigma-Aldrich (Cas No: 62-55-5; Darmstadt, Germany). TAA was freshly prepared in sterile saline. All other chemicals and solvents were of high analytical grade.

### 2.4 UHPLC-ESI-qTOF-MS/MS profiling of TO extracts

UPLC analysis was performed according to the previously described method [24]. About of 2 µL of each sample was injected using partial injection mode, into a Waters ACQUITY I-Class UHPLC system made up of Binary Solvent Module, FL Sample Manager, and separated at a flow rate of 300 µL/min at 55 °C on a Waters ACQUITY UHPLC BEH C-18 column (50 mm length × 2.1 mm i.d, 1.7 µm particle size, Waters GmbH, Eschborn, Germany). The eluting solvents were water (A) and CH_3_CN (B) with formic acid (0.1%) as additive. The elution gradient system was used for chromatographic separation as follows: B (3%, isocratic, 1 min) increasing to 95% B (within 7 min), then at 95% B (3 min), followed by column re-equilibration to 3% B (2.5 min). The column effluents were infused in a hybrid qTOF mass spectrometer (Sciex TripleTOF 6600 LC-MS System, AB Sciex, Darmstadt, Germany) run in negative ion mode with ion spray voltage of −4500 V. The MS experiments were performed at *m*/*z* ranging between 50–1500 in the TOF-scan mode (accumulation time 100 ms). The MS^2^ experiments in information dependent acquisition (IDA) mode (*m*/*z* 50–1500, mass tolerance 25 ppm, intensity ˃ 100, exclude isotope window 4 Da) were accomplished with 50 ms accumulation time at the collision potential (CE) of −40 V, with collision energy spread (CES) of 10 V and declustering potential (DP) of −35 V.

### 2.5 Biological assays

#### 2.5.1 Animals and ethical statement.

Male adult Wistar rats, weighing 150–175 g, were acquired from the National Research Center’s (NRC, Giza, Egypt) animal breeding unit. The rats were housed in conventional settings at a temperature of 25 ◦C, 50% humidity, and a 12:12 dark/light scheme until they were acclimated to the environment for one week prior to any studies. Water and a commercial diet were offered without restriction. As well, water was constantly on hand for the experiment. The study’s methodologies and procedures were carried out in compliance with the Institutional Care and Use Committee (IACUC), National Institutes of Health’s regulations (NIH publication No. 85–23, modified 2011), the National Research Center in Egypt’s Ethics Committee (approval number **Vet CU 25122023835**). In line with the ARRIVE guidelines. Throughout the whole duration of the experiment any necessary steps were carried out to eliminate the rats’ pain, suffering, and loss of weight.

#### 2.5.2 Animal grouping and experimental design.

Forty-eight rats were randomly allocated into 6 groups (8 rats each) as followed: **Group 1**: rats were given saline (i.p.) for 4 weeks that served as normal control group. **Group 2**: served as model group received TAA (200 mg/kg, i.p.) three times per week for 4 weeks [25], **Groups 3 and 4**: rats orally received TO-EtOAC at doses 200 and 400 mg/kg, for 4 weeks, respectively followed by 200 mg/kg i.p. of TAA given three times a week for 4 weeks at a one-hour interval. **Groups 5 and 6**: rats orally received TO-EtOH at doses 200 and 400 mg/kg, for 4 weeks respectively followed by 200 mg/kg i.p. of TAA given three times a week for 4 weeks at a one-hour interval. The treatment started 1 h after TAA injection (200 mg/ kg, i.p.). After TAA injection, supportive therapy (consisting of of 10% glucose mixed with lactate ringer (1 v: 1 v) was given subcutaneously to prevent hypoglycemia and renal failure [26].

From day one until the endpoint of the study, animal well-being was a top priority. A series of proactive and compassionate measures were in place to prevent, alleviate, or eliminate pain and distress whenever possible. Each animal was closely monitored—initially once per day, then twice daily—as the study progressed. A professional laboratory animal technologist conducted these welfare checks, ensuring professional oversight. Key indicators such as respiratory effort and rate were carefully assessed, showing a range from subdued to normal. Observations also included behavior, posture, and mobility, painting a comprehensive picture of each subject’s condition. Individual body weights were recorded daily, providing vital data on health and stability. To monitor core temperatures, a Universal Interface Device (UID) reader was used once or twice per day, offering precise and minimally invasive readings. Prior to scheduled euthanasia, in accordance with humane endpoints, final temperature assessments were conducted using a non-contact infrared thermometer (Lasergrip 774, Etekcity Inc., Anaheim, CA, USA). These readings were taken while gently restraining each animal by the scruff, ensuring minimal stress during the process.

At the ending of the study, all rats were humanely euthanized using an intraperitoneal injection of sodium phenobarbital (40–50 mg/kg), in accordance with established ethical guidelines. Euthanasia was only initiated upon clear behavioral indicators of unrelieved pain—such as a hunched posture, reluctance to move, persistent abdominal licking, or guarding behavior—especially when these symptoms remained despite analgesic treatment (meloxicam, 2 mg/kg, ip). No animals reached an early or unexpected endpoint during the study period. Throughout the entire study, the rats remained in stable health, and no unanticipated mortalities were recorded. Following euthanasia, all biological disposal was conducted under the strict protocols set forth by the National Research Center’s Safety and Health Committee.

The blood samples were collected by retro-orbital puncture. After collection, blood was separated by using a cooling centrifuge (3000 rpm for 15 min, Laboren Zentrifugen, 2K15, Sigma, Germany for separation of serum. The animals were sacrificed at the end of experiment by decapitation under light anesthesia. The liver from each rat was immediately dissected and rinsed with PBS to remove excess blood. The liver was divided into 2 parts: one part was placed in ice-cold phosphate buffer (pH 7.4) to prepare the 20% homogenate that was stored at –80°C. The homogenate was centrifuged for 15 min at 5000 x g using the same centrifuge then the homogenate was used for estimation of liver contents of reduced glutathione (GSH), malondialdehyde (MDA) and tumor necrosis factor- alpha (TNF-α). The second part was placed in 10% formalin and used for histological (H&E) and immunohistochemical assays.

#### 2.5.3 Biochemical assessment.

**2.5.3.1 Determination of liver functions:** Serum levels of alanine aminotransferase (ALT), aspartate aminotransferase (AST), Alkaline phosphatase (ALP) and total bilirubin were evaluated using the commercially available kits (Biodiagnostics, Cairo, Egypt) following manufacturer instructions.

**2.5.3.2 Determination of oxidative stress biomarkers and inflammatory mediators:** Liver homogenate was used for the estimation of MDA, GSH and TNF-α. MDA levels following the method of [27]. GSH level was measured following the protocol of [28]. The hepatic level of TNF-α was determined using a rat (ELISA) kits per the manufacturer’s principles (Sigma-Aldrich, Millipore, Roche. Cat.no. RAB0479)

#### 2.5.4 Histological examination.

Specimens of liver were obtained from different experimental groups then preserved in 10% neutral buffered formalin, routinely processed in water for washing, ascending gradient of alcohol for dehydration followed by clearance in xylene and embedded in paraffin block. Sections were cut using microtome to obtain 4 μm tissue sections that were routinely stained with hematoxylin and eosin stain (H&E) [29]. Assessment of hepatic histopathological injury was performed according to Khalil et al., 2023 [30]. The hepatic lesion score ranged from 0 to 3 (0 = normal, 1 = mild, 2 = moderate and 3 = severe) according to severity of four estimated lesions, that is, fibrosis, necrosis, inflammation and congestion. The total hepatic histological score was achieved by summation of the four parameters. Subsequently, picrosirius red stain was used for fibrous tissue content evaluation and quantification that was examined under bright field and polarized light Leica DM4 B microscope (Wetzlar, Germany). The degree of fibroplasia was measured as area percent using Olympus CellSens dimensions software (Olympus, Tokyo, Japan) [31].

#### 2.5.5 Immunohistochemical assaying.

Hepatic tissue sections among experimental groups were subjected to immunohistochemical analysis of alpha-smooth muscle actin protein (α-SMA) as vital effector marker of tissue fibrogenesis. Firstly, tissue slides were deparaffinized, rehydrated and subjected to thermal-induced antigenic retrieval then endogenous peroxidase blocking using hydrogen peroxide. Afterward, tissue sections were incubated overnight with anti- α-SMA as a primary antibody (monoclonal, ScyTek Laboratories, Inc, USA) in a humid chamber followed by washing using phosphate buffer saline and HRP-labeled secondary antibody was applied at room temperature for two hours. For development of immunoreactivity, chromogen as DAB was used for 10 min and counterstained with Mayer’s hematoxylin. Negative controls were obtained by deletion of incubation with primary antibodies. Positive immunoreactivity was measured and evaluated as area % that were computed randomly in ten stained fields for each group via Cell Sens Olympus software.

### 2.6 Statistical analysis

The standard deviation of the means (n = 8, SD) was used to express the variability of the results. Tukey-Kramer various comparisons were performed for all parametric data after one-way analysis of variance (ANOVA) was used to examine assay results. The non-parametric data were analyzed using Kruskal-Walli’s test, followed by Dunn’s Multiple Comparison test. At *p* < 0.05, the significance threshold was deemed acceptable. GraphPad Prism-8 was used for all statistical experiments (San Diego, CA, USA).

## 3. Results

### 3.1 UHPLC-ESI-qTOF-MS/MS metabolites profiling of *T. oliverianum* extracts

**Fig 1:**
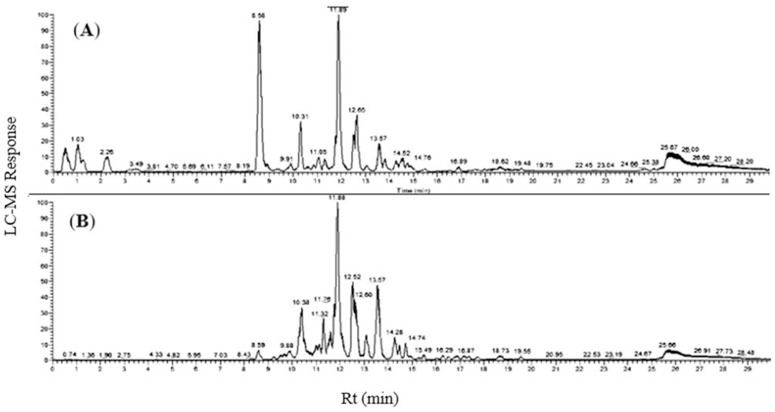
UHPLC-ESI-qTOF-MS/MS base peak chromatograms of *T. oliverianum* above ground parts in negative ionization mode. A) ethanol extract (TO-EtOH) (**A**) and ethyl acetate extract (TO-EtOAc) (**B**).

**Fig 2:**
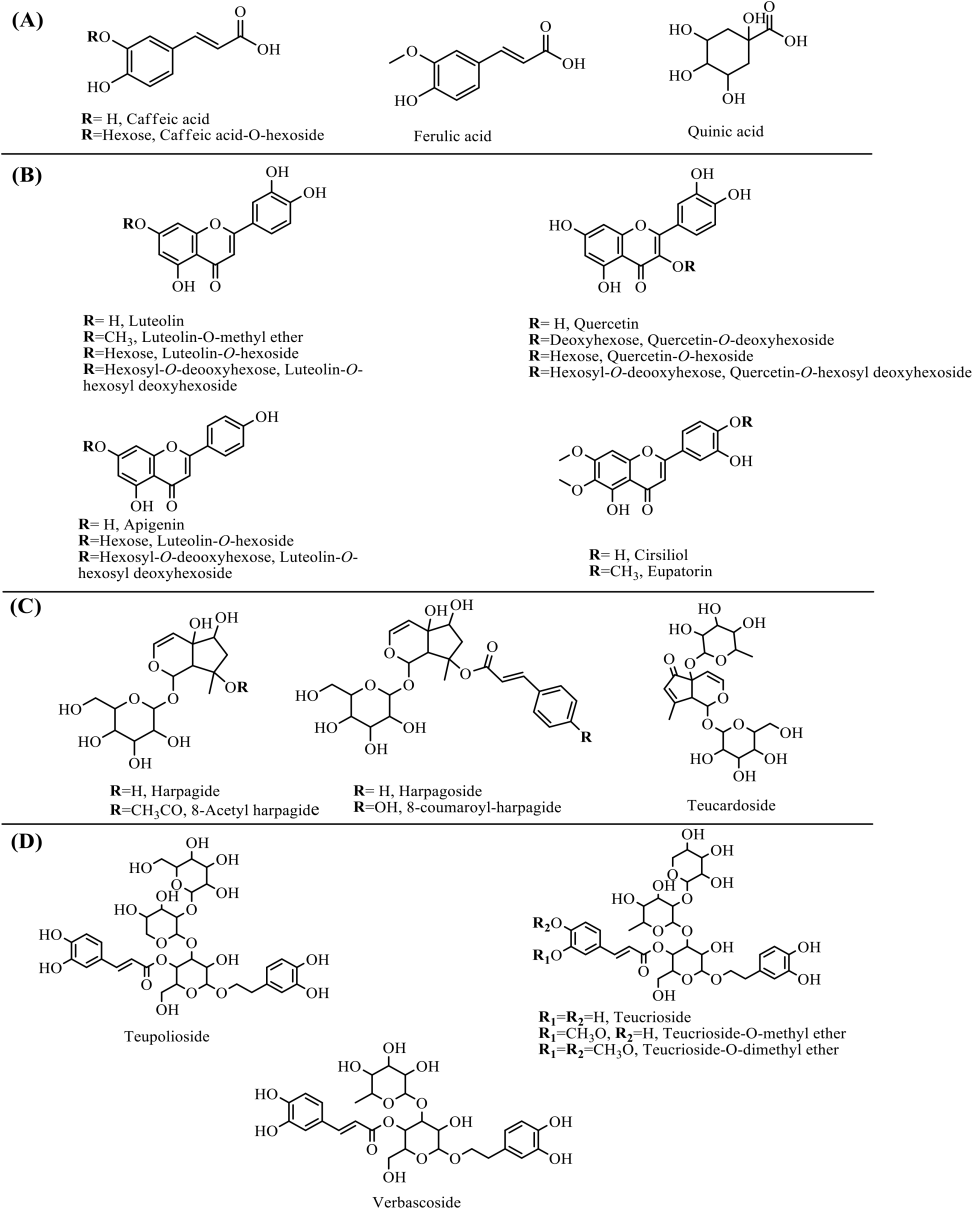
Representative structure of some metabolites identified in *T. oliverianum* above-ground parts *using* UPLC-MS. (**A**) phenolic acids, (**B**) flavonoids/flavonoid glycosides, (**C**) phenylethanoid glycosides, (**D**) iridoid glycosides.

The phytoconstituents of the TO extracts, TO-EtOH and TO-EtOAc, were annotated using UPLC-ESI-HRM/MS platforms [32]. After data processing, a total of 45 and 25 chromatographic peaks were identified in the two extracts, respectively (Fig 1A and 1B). Metabolites’ identification was based on the comparison of retention times, mass spectral data, and fragmentation patterns with previously reported data in the literature. Details of metabolites spectral data are presented in Table 1, arranged according to their retention times in the total ion chromatograms. The identified metabolites belonged to several classes including phenolic acids, flavonoids, phenylethanoid glycosides, iridoid glycosides, and limonoids. Interestingly, flavonoids and phenylethanoid glycosides were most abundant in both TO-EtOH and TO-EtOAc, with several newly reported compounds detected using UPLC-MS analysis. The chemical structures of the predominant metabolites of each class are illustrated in Fig 2. The identification of metabolites in each class are illustrated below in detail.

**Table 1:** UPLC-ESI/MS-MS based characterization of phytoconstituents of the ethanol (EtOH) and ethyl acetate (EtOAc) extracts of *T. oliverianum* above-ground parts in negative ionization mode.

No.	Compound name	R_t_ (min)	Molecular formula	M. Wt.	*m/z* detected and adduct	Error (ppm)	MS^2^ fragments	Chemical class	TO-EtOH	TO-EtOAc
1.	Caffeic acid	0.44	C_9_H_8_O_4_	180	179.0563	1.27	161, 135	Phenolic acid	+	+
2.	Maleic acid	0.53	C_4_H_4_O_4_	115	114.0145	1.30	133	Organic acid	+	+
3.	Caffeic acid-*O*-hexoside	0.75	C_15_H_18_O_9_	342	341.1088	0.75	179, 161, 135	Phenolic glycoside	+	–
4.	Ferulic acid	0.95	C_10_H_10_O_4_	194	193.1088	−4.55	179, 149	Phenolic acid	+	–
5.	Quinic acid	0.97	C_7_H_12_O_6_	192	191.0562	1.15	191, 79, 62	Phenolic acid	+	–
6.	Harpagide	1.06	C_15_H_24_O_10_	364	363.1297	0.94	201, 183, 165	Iridoid glycoside	+	+
7.	Chlorogenic acid	1.67	C_16_H_17_O_9_	354	353.0875	0.78	191, 179	Phenolic acid	+	–
8.	Luteolin-*O*-hexoside	1.76	C_21_H_20_O_11_	448	447.1142	−1.82	285	Flavone glycoside	+	+
9.	*p*-Coumaric acid	3.04	C_9_H_8_O_3_	164	163.0403	1.33	119	Phenolic acid	+	–
10.	Apigenin-*O*-hexoside	3.66	C_21_H_20_O_10_	432	431.1555	1.76	270, 225	Flavone glycoside	+	+
11.	*O*-acetylharpagide	8.51	C_17_H_26_O_11_	406	405.1399	1.93	363, 345	Iridoid glycoside	+	+
12.	Teucardoside	8.68	C_21_H_30_O_19_	490	489.1614	4.09	165, 137	Iridoid glycoside	+	–
13.	Naringenine-*O*-hexosyl-deoxyhexoside	9.11	C_27_H_32_O_14_	580	579.1352	0.74	459, 271	Flavanone glycoside	+	–
14.	Methoxyquercetin-*O*- hexosyl-deoxyhexoside	9.22	C_28_H_32_O_16_	625	624.1401	−2.51	610, 301	Flavonol glycoside	+	+
15.	Quercetin-*O*-deoxyhexoside	9.68	C_21_H_20_O_11_	448	447.1145	1.18	301, 151	Flavonol glycoside	+	–
16.	Teupolioside	9.77	C_35_H_46_O_20_	786	785.2505	3.02	623, 461, 315	Phenylethanoid glycoside	+	–
17.	Teucrioside	9.80	C_34_H_44_O_19_	756	755.2399	3.02	623, 593, 315	Phenylethanoid glycoside	+	+
18.	Verbascoside	9.81	C_29_H_36_O_15_	624	623.1975	0.89	461, 161, 135	Phenylethanoid glycoside	+	+
19.	Quercetin-*O*-hexoside	9.88	C_21_H_20_O_12_	464	463.1662	2.55	427, 301	Flavonol glycoside	+	+
20.	Naringenin	10.06	C_21_H_22_O_10_	272	271.0613	1.18	177, 151	Flavanone	+	–
21.	Castanoside A	10.15	C_34_H_45_O_20_	800	799.2658	2.15	623, 447	Phenylethanoid glycoside	+	+
22.	Unknown	10.29	C_54_H_60_O_32_	1220	1219.3716	−3.56	609, 531, 495, 301	–	+	–
23.	Quercetin-*O*-hexosyl-*O*-deoxyhexoside (Rutin)	10.32	C_27_H_30_O_16_	610	609.1826	1.69	461, 301	Flavonol glycoside	+	+
24.	Quercetin-dimethyl ether-*O*-hexosyl-*O*-deoxyhexoside	10.36	C_29_H_34_O_16_	638	637.1823	0.93	609, 531, 495, 301	Flavonol glycoside	+	+
25.	Teucrioside-*O*-methyl ether	10.52	C_35_H_46_O_19_	770	769.2400	3.70	756, 624, 487	Phenylethanoid glycoside	+	–
26.	Teucrioside-*O*-di-methyl ether	10.65	C_38_H_54_O_19_	784	783.2717	−1.75	770, 756, 624	Phenylethanoid glycoside	+	–
27.	3’-hydroxy 4’, 5,7 trimethoxy flavanone	10.88	C_18_H_18_O_6_	330	329.1549	0.94	313, 301	Flavanone	+	–
28.	Harpagoside	11.26	C_24_H_30_O_11_	494	493.1715	1.04	364, 201, 183, 165	Iridoid glycoside	+	+
29.	Nepetin-*O*-hexoside	11.52	C_22_H_22_O_12_	478	477.2117	−1.10	315, 135	Flavonol glycoside	+	+
30.	Naringenin-*O*-hexoside	11.75	C_21_H_22_O_10_	434	433.1865	0.77	271, 177, 151	Flavanone glycoside	+	+
31.	Koparin-*O*-methyl ether	11.84	C_17_H_14_O_5_	314	313.0433	1.19	300, 286	Methoxyflavone	+	–
32.	Cirsiliol	11.85	C_17_H_14_O_9_	330	329.0667	1.13	314, 300	Methoxy flavone	+	–
33.	Pectolinarigenin	11.96	C_17_H_14_O_6_	314	313.0341	1.01	300, 286	Methoxy flavone	+	+
34.	Khayanthone	12.11	C_32_H_42_O_9_	570	569.2239	1.01		Limonoid	+	–
35.	Unknown	12.17	C_27_H_31_O_15_^+^	595	594.2234	1.33	449, 287	–	+	+
36.	Unknown	12.29	C_15_H_10_O_5_	270	269.2124	4.63	151, 117	–	+	+
37.	Luteolin-*O*-dimethyl ether	12.33	C_17_H_14_O_6_	314	313.0434	4.03	300, 285, 241	Methoxy flavone	+	–
38.	Luteolin-*O*-methyl ether	12.36	C_16_H_12_O_6_	300	299.0574	1.33	285, 241, 151, 133	Methoxy flavone	+	+
39.	Luteolin	12.39	C_15_H_10_O_6_	285	284.0328	4.36	175, 151, 149, 133	Flavone	+	+
40.	Troxerutin	12.41	C_33_H_42_O_19_	742	741.2440	3.93	596, 434	Flavonol glycoside	+	+
41.	Luteolin-*O*-deoxyhexosyl-*O*-hexose	12.45	C_27_H_30_O_15_	594	593.2634	−1.67	477, 285, 241, 151	Flavone glycoside	+	+
42.	*O*-*p*-Coumaroyl-harpagide	12.52	C_24_H_30_O_12_	510	509.2027	0.87	364, 201, 183	Iridoid glycoside	+	+
43.	Selinidin	12.63	C_19_H_20_O_5_	328	327.0587	0.98		Coumarin	+	–
44.	Eupatorin	12.49	C_18_H_16_O_7_	344	343.1552	1.00	328, 313	Methoxy flavone	+	–
45.	Carapin-8(9)-ene	12.81	C_27_H_30_O_7_	466	465	0.77		Liminoid	+	–
46.	Kaempferol-*O*-dehexosyl-*O*-hexoside	13.58	C_27_H_30_O_15_	593	592.2640	4.72	285, 243,199, 151	Flavonol glycoside	+	+
47.	Apigenin-*O*-dehexosyl-*O*-hexoside	13.77	C_27_H_30_O_14_	587	577.2687	4.28	313, 269	Flavone glycoside	+	+
48.	Diosmetin	14.64	C_16_H_12_O_6_	300	299.0441	1.07	284, 256	Methoxy flavone	+	+

*R*_*t*_*:* retention time, ND: Not Identified. TO-EtOH: Ethanol extract; TO-EtOAc: Ethyl acetate extract; + and – denotes the presence or absence of compound in the two extracts.

#### 3.1.1 Phenolics and organic acids.

UPLC-MS analysis led to the identification of 6 phenolic acids (peaks **1**, **3**, **4**, **5**, **7**,& **9**) and one organic acid (peak **2**) in TO-EtOH, belonging to hydroxybenzoates and hydroxycinnamates eluting earlier ranging between 0.44 to 3.04 min, while TO-EtOAc showed only two identified acids including caffeic acid (peak **1**) and maleic acid (peak **2**). Commonly, phenolic acids and their derivatives are identified via the loss of water molecule (18 amu) and carboxylate group (44 amu) in MS/MS spectra [33–35]. Peak **3** m*/z* 341.1088, C_15_H_18_O_9_^-^) was the most prevalent in that class, assigned as caffeic acid-*O*-hexoside showing the loss of hexose moiety (162 amu), in addition to the characteristic fragment ions at *m/z* 179, 161, 135 corresponding to caffeic acid attributed to the loss of hydroxyl and carboxyl groups as shown in S1 Fig. Peaks **4**, **5**, **7**, & **9** were assigned as ferulic acid, quinic acid, chlorogenic acid, and *p*-coumaric acid, respectively as *p*reviously reported in *T. polium* and *T. cavernarum* [36–38]. Meanwhile, all identified phenolic acids were first time to be reported in *T. oliveranium*.

#### 3.1.2 Flavonoids.

Flavonoids represented the major class represented by 25 and 12 peaks in the TO-EtOH and TO-EtOAc, respectively, belonging to flavonoid glycosides, methoxy flavones, and flavanones. Metabolites of this class were identified based on the loss of sugar moieties, methoxy groups (30 amu) and characteristic retro-Diels–Alder (RDA) fragmentation pattern of aglycone. The *O*-glucosyl flavonoids fragmentation yields the most intense fragment corresponding to the aglycone due to loss of sugar moiety [34,35]. The most characteristic flavonoids of *Teucrium* species are quercetin, apigenin and luteolin including 3-, 4′- and 7-O-glycosides as well as some 6-methoxy flavones [39]. Quercetin aglycone (*m/z* 302, C_15_H_10_O_7_) was detected in peaks **14**, **15**, **19**, & **23**, identified as methoxyquercetin-*O*-hexosyl-*O*-deoxyhexoside, quercetin-*O*-deoxyhexoside, quercetin-*O*-hexoside, quercetin-*O*-hexosyl-*O*-deoxyhexoside, and quercetin-dimethyl ether-*O*-rutinoside. As illustrated in S2 Fig, quercetin-*O*-deoxyhexoside (*m/z* 447.1145, C_21_H_20_O_11_^-^) was identified via ^1,5^X-internal cleavage of the pentose sugar to yield fragment ion at *m/z* 343 alongside the loss of whole sugar moiety to yield aglycone at *m/z* 301. Luteolin aglycone (*m/z* 285, C_15_H_9_O_6_) belonging to flavone was found as high abundant ion (derived from homolytic cleavage) in peaks **8**, **38**, **39**, & **42** annotated as luteolin-*O*-hexoside, luteolin-*O*-dimethyl ether, luteolin-*O*-methyl ether, and luteolin-*O*-deoxyhexosyl-*O*-hexose, respectively. As well, two apigenin glycosides were detected in peaks **10** & **48** at *m/z* 431.1555 and 577.2687 yielding fragment ion of apigenin at *m/z* 269 interpreted as apigenin-*O*-hexoside and apigenin-*O*-dehexosyl-*O*-hexoside, respectively. Likewise, cirsiliol and eupatorin were eventually isolated from *T. oliverianum* extracts [8,9]. It should be noted that the glycosidic and aglycone forms of identified flavonoids were abundantly reported in *T. chamaedrys* and *T. polium* [40]. Further, flavanone namely, naringenin has been isolated from *T. chamaedrys* [41].

#### 3.1.3 Phenylethanoid glycosides.

Phenylethanoid glycosides are plant-derived polyphenolics, characterized by cinnamic acid and hydroxyphenylethyl molecules linked with a glucose moiety which form the core of the molecule *via* an ester and glycosidic bonds, respectively [42]. Additionally, the glucose moiety may be attached with other sugars *viz*. rhamnose, xylose, or apiose [43]. Phenylethanoid glycosides have been previously identified in different *Teucrium* species including *T. polium* and *T. chamaedrys* [44,45]. UPLC-MS analysis revealed 6 phenylethanoid glycosides in peaks **16**, **17**, **18**, **21**, **25** & **26**, corresponding to teupolioside, teucrioside, verbascoside, castanoside A, teucrioside-*O*-methyl ether, and teucrioside-*O*-di-methyl ether, respectively in the TO-EtOH, while the TO-EtOAc showed only three peaks (**17**, **18** & **21**) including teucrioside, verbascoside, and castanoside A. As a major compound, teucrioside (*m/z* 755.2399, C_34_H_44_O_19_) was identified based on the loss of pentose (132 amu), and cleavage of an ester linkage to yield two fragment ions at *m/z* 623 and 593, respectively, S3 Fig. Similarly, teupolioside (*m/z* 786, C_35_H_46_O_20_) showed the loss of hexose (163 amu) and cleavage of an ester linkage [46]. Verbascoside (*m/z* 623.1975, C_29_H_36_O_15_^-^) a major phenylpropanoid displayed a principal fragment ion at *m/z* 461 corresponding to the loss of caffeoyl moiety [47]. Methoxylated derivatives of teucrioside in peaks 25 and 26 were detected based on the loss of methoxy group (30 amu). Interestingly, all identified phenylethanoid glycosides were first to be reported in *T. oliverianum*.

#### 3.1.4 Iridoid glycosides.

Iridoids, in form of aglycones or glycosides, represent chemotaxonomic markers of plants belonging to family Lamiaceae and identified in various genera of subfamilies Ajugoideae and Lamioideae [40,48]. Five metabolites in peaks **6**, **11**, **12**, **29**, & **43** were detected as iridoid glycosides in TO-EtOH including harpagide, *O*-acetylharpagide, teucardoside, harpagoside, and *O*-*p*-coumaroyl-har*p*agide, while only harpagide derivatives detected only in TO-EtOAc (peaks **6**, **11**, **29**, & **43**). Harpagide (*m/z* 363.1297, C_15_H_24_O_10_) showed three major fragment ions at *m/z* 201 due to the loss of sugar, *m/z* 185 due to loss of iridoid moiety, *m/z* 165 due to loss of sugar followed by two water molecules [49]. Harpagoside (*m/z* 493.1715, C_24_H_30_O_11_) is a cinnamoyl derivative of harpagide, identified from the loss of cinnamoyl moiety (130 amu), followed by characteristic fragmentation pattern of harpagide. These iridoids are first time to be reported in *T. oliveranium,* while both harpagide and 8-*O*-acetyl-harpagide were previously isolated from *T. chamaedrys* [50].

#### 3.1.5 Limonoids.

Limonoids are classified as tetranortriterpenes to encompass various Furano lactone core structure and to account for several health benefits [51]. Two limonoids were detected in peaks **35** & **46** were detected as limonoids, exclusively in the TO-EtOH extract assigned as khayanthone and carapin-8(9)-ene previously reported in *T. olivarium* [36]. Limonoids were not detected in the TO-EtOAc, and suggestive that for preparing an extract containing this class, ethanol ought to be used as extraction solvent.

### 3.2 Biological assays

#### 3.2.1 Effect of *T. oliverianum* extracts on liver functions.

**Fig 3:**
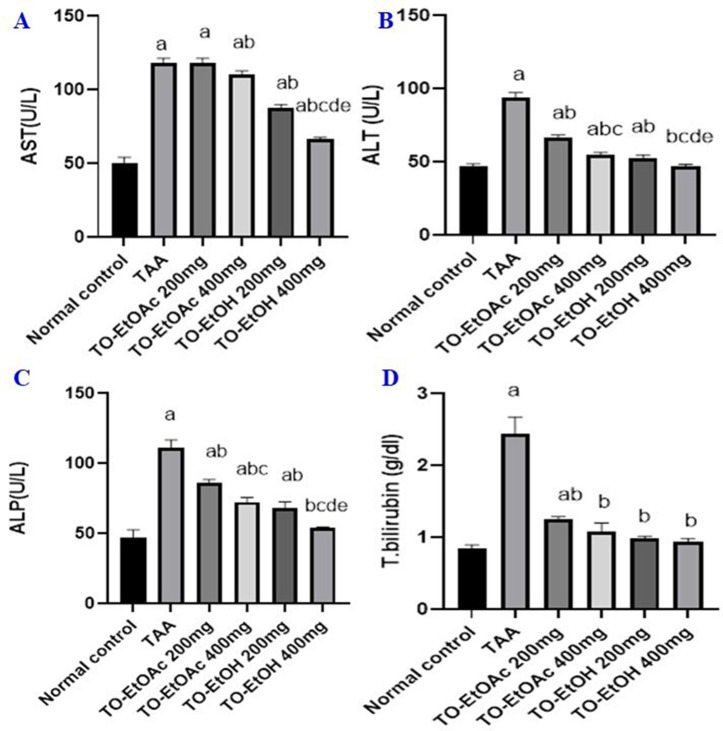
Effect of *T. oliverianum* extracts on liver enzymes and total bilirubin. A) AST, B) ALT, C) ALP D) total bilirubin. Rats were administered thioacetamide (TAA) 200 mg/kg, i.p., or TO-EtOAC at doses (200 and 400 mg/kg) orally, or rats received TO-EtOH at doses (200 and 400 mg/kg) for a period of 4 weeks. The results are expressed as mean ± SD; Significance was tested at p < 0.05 using one-way ANOVA followed by Tukey’s post-hoc test for comparison (n = 8/group). **a** significant difference from control, **b** significant difference from TAA, **c** significant difference from two doses of TO-EtOAc, **d** significant difference from two high doses of different types of extract &**e** significant difference from two doses of TO-EtOH.

As shown in Fig 3, ALT and AST serum levels were significantly increased after i.p. of TAA by 199% and 313% respectively when compared to the normal control group inferring a toxic effect on the liver. In contrast, administration of (200 & 400 mg/ kg) of TO-EtOAC reduced elevated levels of ALT by 69% and 56%, and AST by 74% and 69%, respectively as compared to TAA model group. Oral administration of TO-EtOH (200 & 400 mg/ kg) decreased elevated level of ALT by 54% and 49%, respectively and AST by 55% and 41% respectively (Fig 3A and 3B).

Administration of TAA resulted in a marked increase in serum levels of ALP by 235% compared with normal control rats. ALP level was significantly decreased in TO-EtOAC (200&400 mg/ kg) groups by 76% and 64% respectively, as compared with TAA group. While treatment with (200 &400 mg/ kg) TO-EtOH led to less effect as manifested by decrease serum level of ALP by 60% and 47% respectively (Fig 3C).

Rats receiving TAA (200 mg/kg, i.p.) for 4 weeks were associated with an increase in serum total bilirubin by 289% compared with normal control group. While TO-EtOAC (200&400 mg/ kg) administered rats showed decrease in total bilirubin level by (61% and 43%), respectively when compared to TAA group. As observed in case of ALP, TO-EtOH extract rats in groups 5 and 6 (200 &400 mg/ kg) showed inferior decrease in level of total bilirubin by (39% and 37%) respectively when compared with TAA group (Fig 3D).

#### 3.2.2 Effects of *T. oliverianum* extracts on GSH and MDA hepatic levels.

**Fig 4:**
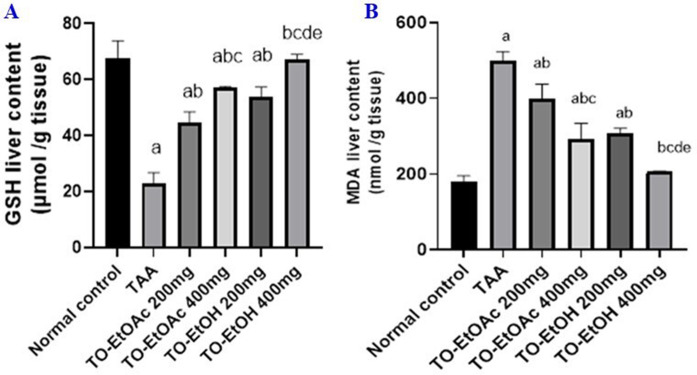
Effects of *T. oliverianum* extracts on hepatic content of GSH and MDA. A) GSH, B) MDA. Rats were administered thioacetamide (TAA) 200 mg/kg, i.p., or TO-EtOAC at doses (200 and 400 mg/kg) orally, or rats received TO-EtOH at doses (200 and 400 mg/kg) for a period of 4 weeks. The results were expressed as mean ± SD; Significance was tested at p < 0.05 using one-way ANOVA followed by Tukey’s post-hoc test for comparison (n = 8/group). **a** significant difference from control, **b** significant difference from TAA, **c** significant difference from two doses of TO-EtOAc, **d** significant difference from two high doses of different types of extract &**e** significant difference from two doses of TO-EtOH.

TAA-induced liver damage demonstrated significant elevation of MDA by 178% concurrent with depletion in GSH level by 66% as compared to normal control group, while animals treated with TO-EtOAc and TO-EtOH extracts 200 & 400 mg/kg demonstrated a significant elevation in GSH by 95%, 150% and 136%, 193% as well as decreased MDA by 20%, 42% and 39%, 59% respectively, compared to TAA- group (Fig 4A and 4B).

#### 3.2.3 Effects of *T. oliverianum* extracts on hepatic content of TNF-*α.*

**Fig 5:**
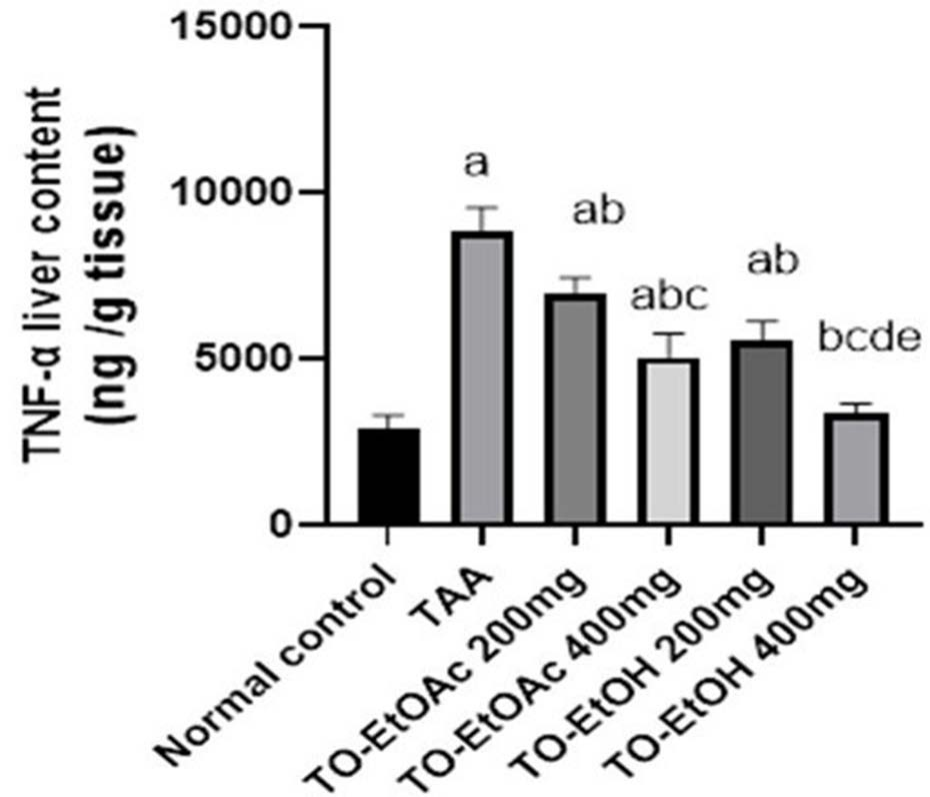
Effect of *T. olivarium* extracts on hepatic content of TNF-*α.* Rats were administered thioacetamide (TAA) 200 mg/kg, i.p., or TO-EtOAC) at doses (200 and 400 mg/kg) orally, or rats received TO-EtOH at doses (200 and 400 mg/kg) for a period of 4 weeks. The results were expressed as mean ± SD; Significance was tested at p < 0.05 using one-way ANOVA followed by Tukey’s post-hoc test for comparison (n = 8/group). **a** significant difference from control, **b** significant difference from TAA, c significant difference from two doses of TO-EtOAc, **d** significant difference from two high doses of different types of extract &**e** significant difference from two doses of TO-EtOH.

TNF-α was found elevated in TAA control group by 200% as compared to normal control group, however, TO-EtOAc and TO-EtOH extracts 200 & 400 mg/kg treatment in both extracts significantly reduced such elevation by 21%, 43% and 37%, 62% respectively, as compared to TAA control group (Fig 5).

#### 3.2.4 Histological and immunohistochemical assays.

**Fig 6:**
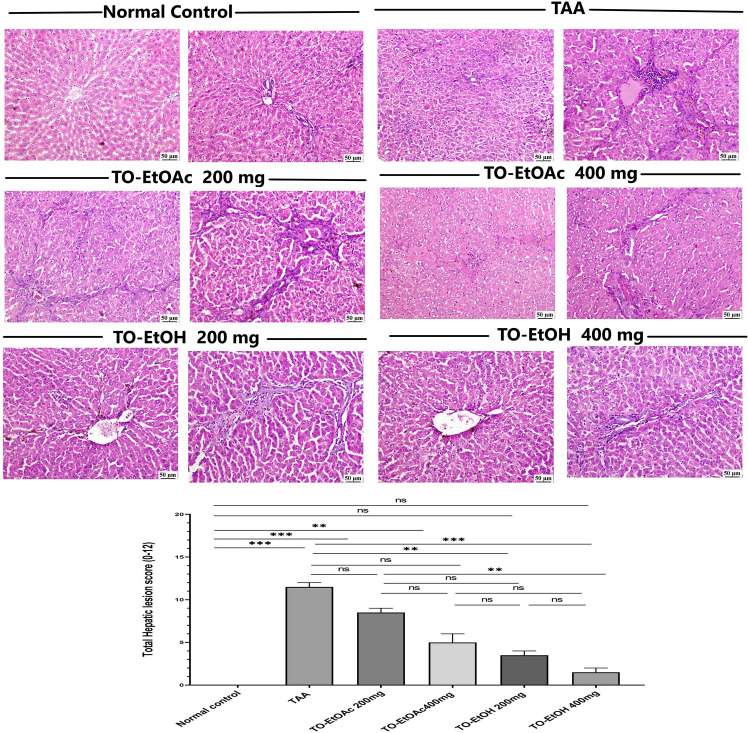
Photographs showing histopathological alterations in hepatic H&E-stained sections among experimental groups. Control group showing normal hepatic cords and hepatic triad, TAA intoxicated group showing hepatocellular degeneration and necrosis with portal hepatitis and fibrosis, TO-EtOAc at doses 200 mg/kg showing portal bridging with focal hepatocellular degeneration and necrosis, TO-EtOAc at doses 400 mg/kg scattered hepatocyte suffered from necrobiotic changes with focal mononuclear inflammatory cells, TO-EtOH at doses 200 mg/kg showing nominal inflammatory cells with mild portal bridging, TO-EtOH at doses 400 mg/kg showing apparent normal hepatic cells and nominal fibroplasia, Bar chart presenting the total hepatic lesion score in different experimental groups. The values were expressed as median with interquartile range that evaluated by the Kruskal-Walli’s test followed by Dunn’s Multiple Comparison test. ^ns^ when P > 0.05, ^**^ when P < 0.01 and ^***^ when P < 0.001.

**3.2.4.1 *T. oliverianum* extracts administration ameliorated hepatic histological changes in TAA- intoxicated rats:** The hepatic sections of the control group revealed normal morphology without any pathological alterations either at the cellular or architectural level. Contrariwise, rats of positive group administrated thioacetamide revealed conspicuous hepatic alterations coincided with prominent hepatocellular degeneration, apoptosis and necrosis in multiple hepatic lobules. The portal areas were heavily infiltrated with mononuclear cells in addition to marked fibroplasia and portal congestion as well as oval cells hyperplasia. Hepatic fibrosis was intensely increased with numerous thick fibrous septa extending from the portal areas toward the parenchyma with portal-to-portal bridging fibrosis causing atrophy of hepatic lobules. Concerning to the examined hepatic sections of treated groups, administration of TO-EtOAc (200 mg/kg) showed mild improvement of hepatic injury indicated by portal fibroplasia that was still restricted to portal triad and not extended toward the hepatic parenchyma. Portal congestion with hyperplasia of biliary epithelium were also noticed accompanied with hepatic necrobiotic changes and portal cellular infiltrates. While rats administrated TO-EtOAc (400 mg/kg) exhibited mild degenerative and necrotic changes of hepatic cells in addition to fibrous strands extending from the portal areas but not connecting the portal areas forming incomplete septa. With regards to TO-EtOH, rats administrated at 200 mg/kg showed improvement at the cellular level of hepatocytes with minimal portal mononuclear cells aggregates with proliferation of biliary epithelium beside newly formed bile ductules. Limited fibrous tissue proliferation at the portal areas was detected causing slight widening without peripheral extension. Administrated TO-EtOH at 400 mg/kg exerted a curative effect that improved hepatic morphology with apparent normal hepatic cells and nominal fibroplasia. In relation to hepatic injury score, rats administrated with thioacetamide recorded the highest lesion score with a significant difference compared with control group (P < 0.001), group of TO-EtOH 200 mg/kg (*P* < 0.01) and 400 mg/kg (*P* < 0.001) (Fig 6). The net histopathology results showed that, in comparison to TAA-intoxicated groups, revealed that both concentrations of TO-EtOAC declined the hepatic injury score while, both concentrations of TO-EtOH groups were distinctly attenuated in the altered hepatic, comparing with TAA intoxicated groups.

**Fig 7:**
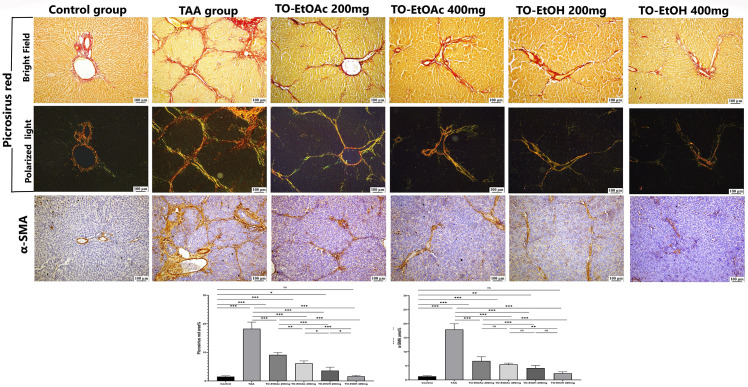
Photomicrographs of hepatic sections stained with picrosirius red (bright field and polarized light) and hepatic immunohistochemical expression of α-SMA in different experimental groups.

**3.2.4.2 Effect of *T. oliverianum* extracts on TAA-induced hepatic fibrosis:** Hepatic fibroplasia was evaluated using picrosirius red stain of collagen as a prominent ECM component. In the control group, normal fibrous tissue was detected around the central vein and portal triad. In contrast, administration of TAA resulted in significantly higher fibrous content that extend periportal and intralobular overall examined hepatic sections. The degree of fibroplasia was diminished among treated groups compared with control positive group (*P* < 0.001). However, TO-EtOAc administration at 200 mg/kg reduced the degree of hepatic fibroplasia, but it was still portal to portal bridging without parenchymal expansion. On the other hand, TO-EtOAc at 400 mg/kg and TO-EtOH at 200 mg/kg revealed incomplete fibrous septa limited to portal triad. Treatment with TO-EtOH at 400 mg/kg substantially declined the hepatic fibroplasia without recording a significant difference with control group (*P* > 0.05) (Fig 7).

****3.2.4.3 Effect of** *T. oliverianum* extracts on α-SMA immunoexpression:** Normal mild expression of α-SMA around central veins and portal triad were observed in hepatic sections of control group. However, livers of TAA intoxicated rats exposed intense α-SMA immunoexpression with thick fibrous septa extending from portal-portal area with parenchymal expansion. The immunoreactivity was significantly inclined in TAA intoxicated rats (*P* < 0.001). The expression was significantly downregulated in treated groups in compared to TAA intoxicated group (*P* < 0.001). In concern to treated groups, the administration of TO-EtOAc recorded no significant difference at both doses (*P* > 0.05). Interestingly, the administration of both doses of TO-EtOH showed a dose dependent down regulation of the immunoreactivity, without significant difference between high dose and control group (*P* > 0.05) (Fig 7).

Lower panel represents fibrosis and α-SMA area percent. Data presented as median with interquartile range that was evaluated using one-way ANOVA followed by Tukey’s multiple comparisons test. ^ns^ when *P* > 0.05, ^*^ when **P* *< 0.05, ^**^ when *P* < 0.01 and ^***^ when **P* *< 0.001.

## 4. Discussion

Hepatic fibrosis is a multicellular reversible injury that is a healing response towards hepatic damage when hepatocytes are replaced by a cellular scar tissue, and extra cellular matrix (ECM) is heavily deposited. The progressive ECM accumulation and intercellular interactions are committed to interfering angiogenesis. The collagen accumulation in the basement membrane affecting the exchange of materials between liver cells, and angiogenesis exerts an inadequate protective mechanism that aid in restoring oxygen supply to the tissue [52].

TAA is one of the most significant hepatotoxicants that is frequently employed in rat models being highly reactive when metabolized, leading to oxidative stress and liver fibrosis [53]. Current study effectively demonstrated the detrimental effects of TAA on the structure and function of the hepatocellular membrane, as revealed by a significant increase in ALT, AST, and ALP as well as a significant increase in serum total bilirubin. It has been extensively demonstrated that the substantial damage to hepatocyte membrane’s integrity and functionality, followed by cellular leakage, is what causes this increase in serum liver enzyme activity [26]. According to present results, administration of TO-EtOAc and TO-ETOH both extracts significantly restored normal levels of AST, ALT, and ALP while also improving serum total bilirubin levels in comparison with TAA intoxicated group. Histological examination revealed that *T. oliverianum* protect the structural integrity of the membranes and hence play a protective function against the damaging effects of TAA on hepatic tissue, provide strong support for its effectiveness. Current findings are consistent with [8] revealing that both the structural organization of the liver and the observed biochemical parameters were significantly improved in the hepatocellular group treated with *T. oliverianum* extracts. These findings are in accordance with that of [54], confirming that in *Teucrium* taxa, *T. polium* is a potent anti-inflammatory and antioxidant agent. The potential *in vivo* antioxidant effects and rich polyphenolic content in *T. polium* ethyl acetate extract have various positive impacts on experimental non-alcoholic steatohepatitis disease. Decrease in serum liver enzyme levels was observed concurrent by improved histological patterns pertaining to lobular inflammation, ballooning degeneration, and steatosis.

Oxidative stress is known as an imbalance between ROS production and antioxidant mechanisms that follow hepatic damage [55]. In the present work, TAA-induced hepatic damage indicated for severe oxidative stress as manifested by a significant elevation in hepatic MDA levels, decrease in GSH level in liver homogenate. Present results are further consistent with the previous study confirming that TAA induced elevation in MDA with a significant decrease in GSH levels [56]. These results indicate a decrease in oxidative stress elicited by TAA upon extracts administration as evidenced by a significant reduction in hepatic MDA content and elevation of GSH levels.

Numerous illnesses, including disorders of the neurological system, hepatic disorders, autoimmune disorders, and others have been linked to ROS and inflammation [57]. ROS trigger the activation of mediator signaling molecules, such as nuclear factor kappa-B, which in turn increases the synthesis of inflammatory cytokines, [58]. Concerning the relationship between liver inflammation and the advancement of fibrosis, injury and damage to the hepatocyte release inflammatory mediators that activate M1 and defense macrophages, which in turn promote the production of proinflammatory cytokines such as IL-6 and tumor necrosis factor-α [59]. TAA, as a hepatotoxin, induces pro-inflammatory and inflammatory cytokines and mediators by macrophages (Kupffer cells) such as TNF-α, that play important role in hepatic inflammation [60]. Administration of *T. oliverianum* extract in the current study improved liver functions, oxidative stress, and liver fibrosis biomarkers, indicating the beneficial effects of extract in regeneration of hepatocytes and posing it, as potential treatment of NASH associated disorders.

There is growing evidence that hepatic stellate cells (HSC) activation is a major factor in liver fibrosis. Following hepatic injury, quiescent HSCs become activated and differentiated into myofibroblasts, expressing high levels of collagen I, III, and α-SMA leading to increased extracellular matrix deposition and liver fibrosis [61]. Therefore, histological findings demonstrated *T. oliverianum* anti-fibrotic ability against TAA- induced liver fibrosis, as evidenced by drop in α-SMA expression. Fibrosis severity is correlated with the expression of the α-SMA protein.

Caballero and his team work [62] revealed the noxious effect of TAA to different cells by the presence of free radicals generated by lipid peroxidation process. Overall, a decrease in the antioxidant defense such as GSH, catalase or SOD, in conjunction with enhanced lipid peroxidation leads to a pro-fibrogenic response [63]. Chronic liver injury is typically associated by prominent activation of sinusoidal stellate cells and portal tract fibroblasts which was evident by induced expression of α-SMA yielding to fibrosis [64]. In contrast, fibroplasia was quietly restricted in the group receiving TO concurrently with TAA, which might be assigned to its anti-proliferative effect on HSC and its ability to block inflammatory infiltration through its anti-inflammatory activities, antioxidant capacity, decrease the lipid peroxidation.

Plant metabolites, especially the polyphenols, have been well characterized as drug treatments in alleviating liver diseases: oxidative stress, metabolism of lipids, resistance to insulin, and inflammatory [65]. Various polyphenols have also been shown to exert protective benefits against liver-toxicity and fibrosis induced by TAA, mostly through their ability to reduce inflammation and oxidative stress.

It has been established that the pharmacological activities of the plant extracts are strappingly correlated with the identified components [66–68]. Generally, many polyphenols, such as flavonoids and their glycosides, as well as some phenolic acids and coumarins, have been documented to prevent liver fibrosis via their ability to inhibit NF-κB and Akt activation might decrease the expression of linked profibrogenesis genes in activated HSCs [65]. Quercetin, as a major representative aglycone of the identified flavonoids (**14**, **15**, **19**, **23** & **24**) showed hepatoprotective activities against different liver diseases, like liver fibrosis, liver steatosis, fatty hepatitis, and liver cancer [69]. Mechanistically, it affects various targets and signaling pathways like fat accumulation inhibition, anti-inflammatory and antioxidant activity, as well as anti-apoptosis [70,71]. Quercetin inhibited liver inflammation via NF-κB/TLR/NLRP3, PI3K/Nrf2-mediated oxidative stress, mTOR activation, and inhibition of apoptotic factors’ expression [72]. At the liver fibrosis stage, it hindered stromal ECM deposition, modifying TGF-1β, endoplasmic reticulum stress (ERs), and apoptosis via regulation of NF-кB/IкBα, p38-MAPK, and Bcl-2/Bax signaling [73]. In TAA-induced liver fibrosis, quercetin decreased liver fibrosis index via provoking HSC apoptosis, and down-expression of MMP-9 and MMP-2 [74]. In in bile duct ligation (BDL) or CCl_4_-induced mice cirrhosis models, quercetin inhibited stellate cell activation and autophagy via TGF-β1/Smads associated with Notch1 signaling pathway [75]. Specifically, quercetin-7-*O*-rhamnoside (**15**), remarkedly ameliorated CCl_4_-induced liver damage in mice represented by enhancement of hepatic GSH content and antioxidant enzymes CAT activities and downregulation of MDA production [76]. Luteolin, as found in many identified peaks (**8**, **37**, **38**, **39** & **41**), was reported to suppress the progression of liver fibrosis in four animal models induced by CCl_4_, DMN, BDL, and thioacetamide directing AKT/mTOR/p70S6K, PI3K/Akt pathways and TGFβ/Smad signaling pathways [77,78]. Further, luteolin-7-*O*-glucoside (**8**) was found to ameliorate the liver damage in GalN/LPS-induced hepatitis via modulation of inflammatory mediators and antioxidants of ICR mice [79]. Another flavonoid, namely naringenin (**20**) displayed preventative activity against dimethylnitrosamine (DMN)-induced liver fibrosis and at doses of 20, 50 and 100 mg/kg mediated through blockage of TGF-β-Smad3 and JNK-Smad3 signaling pathways [80,81]. Also, methoxylated flavonoids such as koparin-*O*-methyl ether (**31**), cirsiliol (**32**), pectolinarigenin (**33**), luteolin-*O*-dimethyl ether (**37**), luteolin-*O*-methyl ether (**38**), eupatorin (**44**) and diosmetin (**48**) were found to modulate the profibrogenic/antifibrogenic balance through suppression of the PI3K/Akt/Smad as well as the PI3K/Akt/mTOR/ribosomal protein S6 kinase 70 kDa (p70S6K) pathways, respectively [82,83].

Regarding the identified phenolic acids; caffeic acid (**1**), ferulic acid (**4**) and chlorogenic acid (**7**), could inhibit HSC activation, and downregulate fibrogenetic factors in CCl_4_-induced liver fibrosis, mediated via inhibiting TLR4/MyD88/NF-*κ*B and TGF-β/Smad signaling pathways as well as various cytokines, including IL-1*β*, IL-6, TNF-*α*, iNOS, and COX-2 [84,85]. Similarly, iridoid glycosides such as harpagide (**6**) displayed significant hepatoprotective effects against heliotrine-induced liver injury manifested by reduced MDA, enhanced SOD activity, and diminished ALT and AST levels [86]. Furthermore, phenylethanoids are reported to downregulate TNF-α and interleukin-1β (IL-1β) protein expression [87,88]. Verbascoside (**18**), as one of the major identified phenylethanoids, exhibited hepatoprotective effect against thioacetamide-induced post-necrotic liver damage [64,89].

The substantial antifibrotic effects of the two plant extracts were predominantly due to the amalgamation and individual actions of these active polyphenolic components. Meanwhile, TO-EtOH was more efficient than TO-EtOAc; this is attributed to the high polarity of ethanol solvent which could extract more polar polyphenolic compounds mainly phenolic acid and flavonoid glycosides that displayed lonely or synergistically in potentiation or inhibition of signaling pathways that control the liver fibrosis cascade. For instance, the TO-EtOAc lacks presence of chlorogenic acid and ferulic acid which were mentioned above as potential downregulators of fibrogenetic factors in the progression of liver fibrosis.

## 5. Conclusion

In conclusion, this study highlights the potential of *T. oliverianum* as a candidate for therapeutic intervention in liver fibrosis. Through a comprehensive metabolite profiling and rigorous biological evaluation, the ethanolic extract emerged as a particularly powerful agent, capable of restoring liver function and architecture disrupted by TAA-induced toxicity. Rich in flavonoids, phenolic acids, and phenylethanoid glycosides, *T. oliverianum* not only validates its traditional medicinal use but also offers a a potential plant-derived candidate for the development of the future antifibrotic therapies. These results lay a strong scientific foundation for continued exploration—where isolating and standardizing its active constituents could unlock new therapeutic frontiers in chronic liver disease management. *T. oliverianum* demonstrates potential as a hepatoprotective agent and may contribute to the development of future therapeutic strategies.

## Supporting information

S1 Fig MS/MS fragmentation pattern of caffeic acid-*O*-hexoside. (DOCX)

S2 Fig MS/MS fragmentation pattern of quercetin-*O*-deoxyhexoside. (DOCX)

S3 Fig MS/MS fragmentation pattern of teucrioside. (DOCX)

## References

[1] Ayoub IM, El-Baset MA, Elghonemy MM, Bashandy SAE, Ibrahim FAA, Ahmed-Farid OAH, et al. Chemical profile of Cyperus laevigatus and its protective effects against thioacetamide-induced hepatorenal toxicity in rats. Molecules. 2022;27(19):6470. doi: 10.3390/molecules27196470 36235007 PMC9573427

[2] Park JU, Kang JH, Rahman MAA, Hussain A, Cho JS, Lee YI. Gastroprotective effects of plants extracts on gastric mucosal injury in experimental sprague-dawley rats. BioMed Res Int. 2019;2019. 10.1155/2019/8759708 PMC6398063 30906783

[3] Asnaashari S, Dastmalchi S, Javadzadeh Y. Gastroprotective effects of herbal medicines (roots). Int J Food Prop. 2018;21(1):902–20. doi: 10.1080/10942912.2018.1473876

[4] Elshamy AI, Abdallah HM, Farrag ARH, Riciputi Y, Pasini F, Taher RF. Artichoke phenolics confer protection against acute kidney injury. Rev Bras Farmacogn. 2020;30:34–42.

[5] Abdullah FO, Hussain FHS, Sardar AS, Gilardoni G, Thu ZM, Vidari G. Bio-active compounds from Teucrium plants used in the traditional medicine of Kurdistan region, Iraq. Molecules. 2022;27(10):3116. doi: 10.3390/molecules27103116 35630593 PMC9145536

[6] Candela RG, Rosselli S, Bruno M, Fontana G. A review of the phytochemistry, traditional uses and biological activities of the essential oils of genus Teucrium. Planta Medica. 2020;87(06):432–79. 33296939 10.1055/a-1293-5768

[7] Al-Yahya MA, El-Feraly FS, Dunbar DC, Muhammad I. neo-Clerodane diterpenoids from Teucrium oliverianum and structure revision of teucrolin E. Phytochemistry. 2002;59(4):409–14. doi: 10.1016/s0031-9422(01)00396-x 11830158

[8] Shahat AA, Alsaid MS, Khan JA, Higgins M, Dinkova-Kostova AT. Chemical constituents and NAD (P) H: quinone oxidoreductase 1 (NQO1) inducer activity of Teucrium oliverianum Ging. ex Benth. IJTK. 2016;15(2):232–6.

[9] Al-Yahya MA, Muhammad I, Mirza HH, El-Feraly FS, McPhail AT. Neocleordane diterpenoids and their artifacts from Teucrium olivarianum. J Nat Prod. 1993;56(6):830–42.

[10] Sadeghi Z, Yang J-L, Venditti A, Moridi Farimani M. A review of the phytochemistry, ethnopharmacology and biological activities of Teucrium genus (Germander). Nat Prod Res. 2022;36(21):5647–64. doi: 10.1080/14786419.2021.2022669 34986708

[11] Arzi A, Namjouyan F, Sarahroodi S, Khorasgani ZN, Macvandi E. The study of antinociceptive effect of hydroalcoholic extract of Teucrium oliverianum (a plant used in southern Iranian traditional medicine) in rat by formalin test. Pak J Biol Sci. 2011;14(23):1066–9. doi: 10.3923/pjbs.2011.1066.1069 22590841

[12] Kim WR, Brown RSJ, Terrault NA, El‐Serag H. Burden of liver disease in the United States: summary of a workshop. Hepatology. 2002;36(1):227–42. 12085369 10.1053/jhep.2002.34734

[13] Stickel F, Schuppan D. Herbal medicine in the treatment of liver diseases. Dig Liver Dis. 2007;39(4):293–304. doi: 10.1016/j.dld.2006.11.004 17331820

[14] Avraham Y, Grigoriadis NC, Magen I, Poutahidis T, Vorobiav L, Zolotarev O, et al. Capsaicin affects brain function in a model of hepatic encephalopathy associated with fulminant hepatic failure in mice. Br J Pharmacol. 2009;158(3):896–906. doi: 10.1111/j.1476-5381.2009.00368.x 19764982 PMC2765608

[15] Mousa AA, El-Gansh HAI, Eldaim MAA, Mohamed MAE-G, Morsi AH, El Sabagh HS. Protective effect of Moringa oleifera leaves ethanolic extract against thioacetamide-induced hepatotoxicity in rats via modulation of cellular antioxidant, apoptotic and inflammatory markers. Environ Sci Pollut Res. 2019;26:32488–504. 10.1007/s11356-019-06368-4 31617137

[16] Yogalakshmi B, Viswanathan P, Anuradha CV. Investigation of antioxidant, anti-inflammatory and DNA-protective properties of eugenol in thioacetamide-induced liver injury in rats. Toxicology. 2010;268(3):204–12. doi: 10.1016/j.tox.2009.12.018 20036707

[17] Wang ME, Chen YC, Chen IS, Hsieh SC, Chen SS, Chiu CH. Curcumin protects against thioacetamide-induced hepatic fibrosis by attenuating the inflammatory response and inducing apoptosis of damaged hepatocytes. J Nutr Biochem. 2012;23(10):1352–66. 22221674 10.1016/j.jnutbio.2011.08.004

[18] Hajovsky H, Hu G, Koen Y, Sarma D, Cui W, Moore DS, et al. Metabolism and toxicity of thioacetamide and thioacetamide S-oxide in rat hepatocytes. Chem Res Toxicol. 2012;25(9):1955–63. doi: 10.1021/tx3002719 22867114 PMC3444651

[19] Alshawsh MA, Abdulla MA, Ismail S, Amin ZA. Hepatoprotective effects of Orthosiphon stamineus extract on thioacetamide-induced liver cirrhosis in rats. Evid Based Complement Altern Med. 2011;2011. 10.1155/2011/103039 PMC3106356 21647311

[20] Pérez-Tortosa V, López-Orenes A, Martínez-Pérez A, Ferrer MA, Calderón AA. Antioxidant activity and rosmarinic acid changes in salicylic acid-treated Thymus membranaceus shoots. Food Chem. 2012;130(2):362–9. doi: 10.1016/j.foodchem.2011.07.051

[21] Gupta NK, Dixit VK. Hepatoprotective activity of Cleome viscosa Linn. extract against thioacetamide-induced hepatotoxicity in rats. Nat Prod Res. 2009;23(14):1289–97. doi: 10.1080/14786410802447302 19735042

[22] Rui L, Silva E, Silva T, Portela TCL, Silva A, Cogliati B. Cirrhosis in rats does not resolve in the long-term after induction by thioacetamide model. J Morphol Sci. 2014;31(01):033–41.

[23] Chaudhary SA, al-Waṭanīyah M. Flora of the Kingdom of Saudi Arabia: illustrated; 2001.

[24] Otify AM, Ibrahim RM, Abib B, Laub A, Wessjohann LA, Jiang Y, et al. Unveiling metabolome heterogeneity and new chemicals in 7 tomato varieties via multiplex approach of UHPLC-MS/MS, GC-MS, and UV-Vis in relation to antioxidant effects as analyzed using molecular networking and chemometrics. Food Chem. 2023;417:135866. doi: 10.1016/j.foodchem.2023.135866 36913868

[25] Alkreathy HM, Esmat A. Lycorine ameliorates thioacetamide-induced hepatic fibrosis in rats: emphasis on antioxidant, anti-inflammatory, and STAT3 inhibition effects. Pharmaceuticals (Basel). 2022;15(3):369. doi: 10.3390/ph15030369 35337166 PMC8955817

[26] Baraka SM, Mowaad NA, Ibrahim S, Korany RM, El-Sayed AF, Hassan AA. Green synthesized cerium oxide nanoparticles ameliorate hepatic and cognitive dysfunctions in thioacetamide-induced hepatic encephalopathy in rats: modulation of TLR-4/NF-κB/Caspase-3 signaling pathways. J Drug Deliv Sci Technol. 2023;87:104846.

[27] Matsumiya H, Hoshino H. Selective determination of beryllium(II) ion at picomole per decimeter cubed levels by kinetic differentiation mode reversed-phase high-performance liquid chromatography with fluorometric detection using 2-(2’-hydroxyphenyl)-10-hydroxybenzo[H]quinoline as precolumn chelating reagent. Anal Chem. 2003;75(3):413–9. doi: 10.1021/ac0260847 12585465

[28] Vaziri ND, Wang XQ, Oveisi F, Rad B. Induction of oxidative stress by glutathione depletion causes severe hypertension in normal rats. Hypertension. 2000;36(1):142–6. doi: 10.1161/01.hyp.36.1.142 10904027

[29] Bancroft JD, Gamble M. Theory and practice of histological techniques. Elsevier Health Sciences; 2008.

[30] Khalil HM, Khalil IA, Al-Mokaddem AK, Hassan M, El-Shiekh RA, Eliwa HA. Ashwagandha-loaded nanocapsules improved the behavioral alterations, and blocked MAPK and induced Nrf2 signaling pathways in a hepatic encephalopathy rat model. Drug Deliv Transl Res. 2023;13(1):252–74. 35672652 10.1007/s13346-022-01181-y PMC9726678

[31] Husain H, Latief U, Ahmad R. Pomegranate action in curbing the incidence of liver injury triggered by diethylnitrosamine by declining oxidative stress via Nrf2 and NFκB regulation. Sci Rep. 2018;8(1):8606. doi: 10.1038/s41598-018-26524-9 29872102 PMC5988808

[32] El-Nashar HAS, Taleb M, El-Shazly M, Zhao C, Farag MA. Polysaccharides (pectin, mucilage, and fructan inulin) and their fermented products: a critical analysis of their biochemical, gut interactions, and biological functions as antidiabetic agents. Phytother Res. 2024;38(2):662–93. doi: 10.1002/ptr.8067 37966040

[33] Farag MA, El-Kersh DM, Ehrlich A, Choucry MA, El-Seedi H, Frolov A, et al. Variation in Ceratonia siliqua pod metabolome in context of its different geographical origin, ripening stage and roasting process. Food Chem. 2019;283:675–87. doi: 10.1016/j.foodchem.2018.12.118 30722926

[34] Abdelghffar EA, El-Nashar HAS, Al-Mohammadi AGA, Eldahshan OA. Orange fruit (Citrus sinensis) peel extract attenuates chemotherapy-induced toxicity in male rats. Food Funct. 2021;12(19):9443–55. doi: 10.1039/d1fo01905h 34606555

[35] Otify AM, El-Sayed AM, Michel CG, Farag MA. Metabolites profiling of date palm (Phoenix dactylifera L.) commercial by-products (pits and pollen) in relation to its antioxidant effect: a multiplex approach of MS and NMR metabolomics. Metabolomics. 2019;15(9):119. doi: 10.1007/s11306-019-1581-7 31456052

[36] Noumi E, Snoussi M, Anouar EH, Alreshidi M, Veettil VN, Elkahoui S, et al. HR-LCMS-based metabolite profiling, antioxidant, and anticancer properties of Teucrium polium L. Methanolic extract: computational and in vitro study. Antioxidants (Basel). 2020;9(11):1089. doi: 10.3390/antiox9111089 33167507 PMC7694502

[37] Göğer F, Kaya A, Dinç M, Doğu SD. Phenolic compounds determination and antioxidant activity of Teucrium cavernarum. Eskişeh Tek Üniv Bilim Teknol Derg - C Yaşam Bilim Biyol. 2019;8(2):229–37. doi: 10.18036/estubtdc.599094

[38] Stanković MS, Stefanović O, Čomić L, Topuzović M, Radojević I, Solujić S. Antimicrobial activity, total phenolic content and flavonoid concentrations of Teucrium species. Cent Eur J Biol. 2012;7:664–71.

[39] Noumi E, Snoussi M, Anouar EH, Alreshidi M, Veettil VN, Elkahoui S, et al. HR-LCMS-based metabolite profiling, antioxidant, and anticancer properties of Teucrium polium L. Methanolic extract: computational and in vitro study. Antioxidants (Basel). 2020;9(11):1089. doi: 10.3390/antiox9111089 33167507 PMC7694502

[40] Abdullah FO, Hussain FHS, Sardar AS, Gilardoni G, Thu ZM, Vidari G. Bio-active compounds from Teucrium plants used in the traditional medicine of Kurdistan region, Iraq. Molecules. 2022;27(10):3116. doi: 10.3390/molecules27103116 35630593 PMC9145536

[41] Tariq M, Ageel AM, al-Yahya MA, Mossa JS, al-Said MS. Anti-inflammatory activity of Teucrium polium. Int J Tissue React. 1989;11(4):185–8. 2634627

[42] Jiménez C, Riguera R. Phenylethanoid glycosides in plants: structure and biological activity. Nat Prod Rep. 1994;11(6):591–606. doi: 10.1039/np9941100591 15209134

[43] Wu L, Georgiev MI, Cao H, Nahar L, El-Seedi HR, Sarker SD, et al. Therapeutic potential of phenylethanoid glycosides: a systematic review. Med Res Rev. 2020;40(6):2605–49. doi: 10.1002/med.21717 32779240

[44] Antognoni F, Iannello C, Mandrone M, Scognamiglio M, Fiorentino A, Giovannini PP, et al. Elicited Teucrium chamaedrys cell cultures produce high amounts of teucrioside, but not the hepatotoxic neo-clerodane diterpenoids. Phytochemistry. 2012;81:50–9. doi: 10.1016/j.phytochem.2012.05.027 22769437

[45] Elmasri WA, Yang T, Tran P, Hegazy M-EF, Hamood AN, Mechref Y, et al. Teucrium polium phenylethanol and iridoid glycoside characterization and flavonoid inhibition of biofilm-forming Staphylococcus aureus. J Nat Prod. 2015;78(1):2–9. doi: 10.1021/np5004092 25524452

[46] Oganesyan GB, Galstyan AM, Mnatsakanyan VA, Shashkov AS, Agababyan PV. Phenylpropanoid glycosides of Teucrium polium. Chem Nat Compd. 1991;27(5):556–9. doi: 10.1007/bf00630353

[47] Plaza A, Montoro P, Benavides A, Pizza C, Piacente S. Phenylpropanoid glycosides from Tynanthus panurensis: characterization and LC-MS quantitative analysis. J Agric Food Chem. 2005;53(8):2853–8. doi: 10.1021/jf0479867 15826030

[48] Frezza C, Venditti A, Matrone G, Serafini I, Foddai S, Bianco A, et al. Iridoid glycosides and polyphenolic compounds from Teucrium chamaedrys L. Nat Prod Res. 2018;32(13):1583–9. doi: 10.1080/14786419.2017.1392948 29058476

[49] Colas C, Garcia P, Popot M-A, Bonnaire Y, Bouchonnet S. Liquid chromatography/electrospray ionization mass spectrometric characterization of Harpagophytum in equine urine and plasma. Rapid Commun Mass Spectrom. 2006;20(22):3257–66. doi: 10.1002/rcm.2721 17044124

[50] Frezza C, Venditti A, Matrone G, Serafini I, Foddai S, Bianco A, et al. Iridoid glycosides and polyphenolic compounds from Teucrium chamaedrys L. Nat Prod Res. 2018;32(13):1583–9. doi: 10.1080/14786419.2017.1392948 29058476

[51] Luo J, Sun Y, Li Q, Kong L. Research progress of meliaceous limonoids from 2011 to 2021. Nat Prod Rep. 2022;39(6):1325–65. doi: 10.1039/d2np00015f 35608367

[52] Tsuchida T, Friedman SL. Mechanisms of hepatic stellate cell activation. Nat Rev Gastroenterol Hepatol. 2017;14(7):397–411. doi: 10.1038/nrgastro.2017.38 28487545

[53] Ebaid H, Bashandy SAE, Morsy FA, Al-Tamimi J, Hassan I, Alhazza IM. Protective effect of gallic acid against thioacetamide-induced metabolic dysfunction of lipids in hepatic and renal toxicity. J King Saud Univ Sci. 2023;35(3):102531. doi: 10.1016/j.jksus.2022.102531

[54] Amini R, Yazdanparast R. Suppression of hepatic TNF-α and TGF-β gene expressions in rats with induced nonalcoholic steatohepatitis. Pharmacology Online. 2009;3:340–50.

[55] El-Mihi KA, Kenawy HI, El-Karef A, Elsherbiny NM, Eissa LA. Naringin attenuates thioacetamide-induced liver fibrosis in rats through modulation of the PI3K/Akt pathway. Life Sci. 2017;187:50–7. doi: 10.1016/j.lfs.2017.08.019 28830755

[56] AbouSamra MM, Elgohary R, Mansy SS. Innovated pirfenidone loaded lecithin nanocapsules for targeting liver fibrosis: formulation, characterization and in vivo study. Int J Pharm. 2023;631:122539. 36572266 10.1016/j.ijpharm.2022.122539

[57] Sánchez A, Calpena AC, Clares B. Evaluating the oxidative stress in inflammation: role of melatonin. Int J Mol Sci. 2015;16(8):16981–7004. doi: 10.3390/ijms160816981 26225957 PMC4581180

[58] Naik E, Dixit VM. Mitochondrial reactive oxygen species drive proinflammatory cytokine production. J Exp Med. 2011;208(3):417–20. doi: 10.1084/jem.20110367 21357740 PMC3058577

[59] Li H, You H, Fan X, Jia J. Hepatic macrophages in liver fibrosis: pathogenesis and potential therapeutic targets. BMJ Open Gastroenterol. 2016;3(1):e000079. doi: 10.1136/bmjgast-2016-000079 27252881 PMC4885270

[60] El Awdan SA, Abdel Rahman RF, Ibrahim HM, Hegazy RR, El Marasy SA, Badawi M, et al. Regression of fibrosis by cilostazol in a rat model of thioacetamide-induced liver fibrosis: up regulation of hepatic cAMP, and modulation of inflammatory, oxidative stress and apoptotic biomarkers. PLoS One. 2019;14(5):e0216301. doi: 10.1371/journal.pone.0216301 31067255 PMC6505801

[61] Jiao W, Bai M, Yin H, Liu J, Sun J, Su X, et al. Therapeutic effects of an inhibitor of thioredoxin reductase on liver fibrosis by inhibiting the transforming growth factor-β1/Smads pathway. Front Mol Biosci. 2021;8:690170. doi: 10.3389/fmolb.2021.690170 34540892 PMC8440796

[62] Caballero ME, Berlanga J, Ramirez D, Lopez-Saura P, Gozalez R, Floyd DN, et al. Epidermal growth factor reduces multiorgan failure induced by thioacetamide. Gut. 2001;48(1):34–40. doi: 10.1136/gut.48.1.34 11115820 PMC1728178

[63] Mormone E, George J, Nieto N. Molecular pathogenesis of hepatic fibrosis and current therapeutic approaches. Chem Biol Interact. 2011;193(3):225–31. doi: 10.1016/j.cbi.2011.07.001 21803030 PMC3171510

[64] Zimmermann HW, Seidler S, Gassler N, Nattermann J, Luedde T, Trautwein C, et al. Interleukin-8 is activated in patients with chronic liver diseases and associated with hepatic macrophage accumulation in human liver fibrosis. PLoS One. 2011;6(6):e21381. doi: 10.1371/journal.pone.0021381 21731723 PMC3120868

[65] Li S, Tan HY, Wang N, Cheung F, Hong M, Feng Y. The potential and action mechanism of polyphenols in the treatment of liver diseases. Oxid Med Cell Longev. 2018;2018:8394818. doi: 10.1155/2018/8394818 29507653 PMC5817364

[66] Younis T, Jabeen F, Hussain A, Rasool B, Raza Ishaq A, Nawaz A, et al. Antioxidant and pulmonary protective potential of Fraxinus xanthoxyloides bark extract against CCl4 -induced toxicity in rats. Chem Biodivers. 2023;20(3):e202200755. doi: 10.1002/cbdv.202200755 36722706

[67] Rabie O, El-Nashar HAS, Majrashi TA, Al-Warhi T, El Hassab MA, Eldehna WM, et al. Chemical composition, seasonal variation and antiaging activities of essential oil from Callistemon subulatus leaves growing in Egypt. J Enzyme Inhib Med Chem. 2023;38(1):2224944. doi: 10.1080/14756366.2023.2224944 37369580 PMC10304439

[68] Jamaddar S, Sarkar C, Akter S, Mubarak MS, El-Nashar HAS, El-Shazly M, et al. Brazilin: an updated literature-based review on its promising therapeutic approaches and toxicological studies. S Afr J Bot. 2023;158:118–32. doi: 10.1016/j.sajb.2023.04.053

[69] Zhao X, Wang J, Deng Y, Liao L, Zhou M, Peng C, et al. Quercetin as a protective agent for liver diseases: a comprehensive descriptive review of the molecular mechanism. Phytother Res. 2021;35(9):4727–47. doi: 10.1002/ptr.7104 34159683

[70] Chen Z, Huang C, Ma T, Jiang L, Tang L, Shi T, et al. Reversal effect of quercetin on multidrug resistance via FZD7/β-catenin pathway in hepatocellular carcinoma cells. Phytomedicine. 2018;43:37–45. doi: 10.1016/j.phymed.2018.03.040 29747752

[71] Schwingel TE, Klein CP, Nicoletti NF, Dora CL, Hadrich G, Bica CG, et al. Effects of the compounds resveratrol, rutin, quercetin, and quercetin nanoemulsion on oxaliplatin-induced hepatotoxicity and neurotoxicity in mice. Naunyn Schmiedebergs Arch Pharmacol. 2014;387(9):837–48. doi: 10.1007/s00210-014-0994-0 24908156

[72] El-Shawi OE, El-Nashar HAS, Abd El-Rahman SS, Eldahshan OA, Singab ANB. Protective effect of acrocarpus fraxinifolius extract against hepatic fibrosis induced by Gamma irradiation and carbon tetrachloride in albino rats. Int J Radiat Biol. 2023;99(2):270–80. doi: 10.1080/09553002.2022.2087926 35675546

[73] Wang R, Zhang H, Wang Y, Song F, Yuan Y. Inhibitory effects of quercetin on the progression of liver fibrosis through the regulation of NF-кB/IкBα, p38 MAPK, and Bcl-2/Bax signaling. Int Immunopharmacol. 2017;47:126–33. doi: 10.1016/j.intimp.2017.03.029 28391159

[74] Afifi NA, Ibrahim MA, Galal MK. Hepatoprotective influence of quercetin and ellagic acid on thioacetamide-induced hepatotoxicity in rats. Can J Physiol Pharmacol. 2018;96(6):624–9. doi: 10.1139/cjpp-2017-0651 29414242

[75] Wu L, Zhang Q, Mo W, Feng J, Li S, Li J, et al. Quercetin prevents hepatic fibrosis by inhibiting hepatic stellate cell activation and reducing autophagy via the TGF-β1/Smads and PI3K/Akt pathways. Sci Rep. 2017;7(1):9289. doi: 10.1038/s41598-017-09673-5 28839277 PMC5571156

[76] Huang Z-Q, Chen P, Su W-W, Wang Y-G, Wu H, Peng W, et al. Antioxidant activity and hepatoprotective potential of quercetin 7-rhamnoside in vitro and in vivo. Molecules. 2018;23(5):1188. doi: 10.3390/molecules23051188 29772655 PMC6100316

[77] Li J, Li X, Xu W, Wang S, Hu Z, Zhang Q, et al. Antifibrotic effects of luteolin on hepatic stellate cells and liver fibrosis by targeting AKT/mTOR/p70S6K and TGFβ/Smad signalling pathways. Liver Int. 2015;35(4):1222–33. doi: 10.1111/liv.12638 25040634

[78] Alamri ZZ. Effect of luteolin and quercetin on thioacetamide induced hepatic fibrosis in rats. Int J Pharmacol. 2019;15(7):863–71.

[79] Park CM, Song Y-S. Luteolin and luteolin-7-O-glucoside protect against acute liver injury through regulation of inflammatory mediators and antioxidative enzymes in GalN/LPS-induced hepatitic ICR mice. Nutr Res Pract. 2019;13(6):473–9. doi: 10.4162/nrp.2019.13.6.473 31814922 PMC6883227

[80] Hernández-Aquino E, Zarco N, Casas-Grajales S, Ramos-Tovar E, Flores-Beltrán RE, Arauz J, et al. Naringenin prevents experimental liver fibrosis by blocking TGFβ-Smad3 and JNK-Smad3 pathways. World J Gastroenterol. 2017;23(24):4354–68. doi: 10.3748/wjg.v23.i24.4354 28706418 PMC5487499

[81] Hernández-Aquino E, Quezada-Ramírez MA, Silva-Olivares A, Casas-Grajales S, Ramos-Tovar E, Flores-Beltrán RE. Naringenin attenuates the progression of liver fibrosis via inactivation of hepatic stellate cells and profibrogenic pathways. Eur J Pharmacol. 2019;865:172730. 31618621 10.1016/j.ejphar.2019.172730

[82] Yu D k, Zhang C x, Zhao S s, Zhang S h, Zhang H, Cai S y. The anti-fibrotic effects of epigallocatechin-3-gallate in bile duct-ligated cholestatic rats and human hepatic stellate LX-2 cells are mediated by the PI3K/Akt/Smad pathway. Acta Pharmacol Sin. 2015;36(4):473–82. 25832428 10.1038/aps.2014.155 PMC4387300

[83] Wang Q, Wen R, Lin Q, Wang N, Lu P, Zhu X. Wogonoside shows antifibrotic effects in an experimental regression model of hepatic fibrosis. Dig Dis Sci. 2015;60(11):3329–39. doi: 10.1007/s10620-015-3751-4 26130019

[84] Shi H, Dong L, Jiang J, Zhao J, Zhao G, Dang X, et al. Chlorogenic acid reduces liver inflammation and fibrosis through inhibition of toll-like receptor 4 signaling pathway. Toxicology. 2013;303:107–14. doi: 10.1016/j.tox.2012.10.025 23146752

[85] Reyes MT, Mourelle M, Hong E, Muriel P. Caffeic acid prevents liver damage and ameliorates liver fibrosis induced by CCI4 in the rat. Drug Dev Res. 1995;36(3):125–8.

[86] Singh B, Kumar S, Anal JMH, Surmal O, Bhat MN, Prasad M. Biological and chemical aspects of the genus Ajuga L. In: Bioactives and pharmacology of Lamiaceae. Apple Academic Press; 2024. p. 1–27.

[87] Zhang M-Q, Ren X, Zhao Q, Yue S-J, Fu X-M, Li X, et al. Hepatoprotective effects of total phenylethanoid glycosides from Acanthus ilicifolius L. against carbon tetrachloride-induced hepatotoxicity. J Ethnopharmacol. 2020;256:112795. doi: 10.1016/j.jep.2020.112795 32224197

[88] Ahmed S, Khan ST, Zargaham MK, Khan AU, Khan S, Hussain A, et al. Potential therapeutic natural products against Alzheimer’s disease with Reference of Acetylcholinesterase. Biomed Pharmacother. 2021;139:111609. doi: 10.1016/j.biopha.2021.111609 33915501

[89] Jaramillo-Morales OA, Díaz-Cervantes E, Via LD, Garcia-Argaez AN, Espinosa-Juárez JV, Ovando-Zambrano JC. Hepatoprotective activity, in silico analysis, and molecular docking study of verbascoside from Leucophyllum frutescens in rats with post-necrotic liver damage. Scientia Pharmaceutica. 2023;91(3).

