# Supplementary figures and images for: Phenolic-rich extracts of Teucrium oliverianum confer protection against thioacetamide-induced liver fibrosis in rats: Insights from metabolomics, biochemical and histopathological analysis
Source: PLoS One. 2025 Sep 2;20(9):e0330595. doi: 10.1371/journal.pone.0330595 (PMC12404504; doi:10.1371/journal.pone.0330595)

**
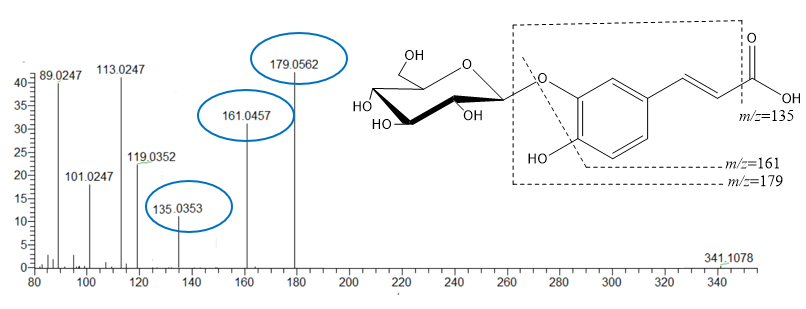
**

**Figure S1.** MS/MS fragmentation pattern of caffeic acid-*O*-hexoside

Supplement: S1 Fig — (DOCX) [file pone.0330595.s001.docx]

**
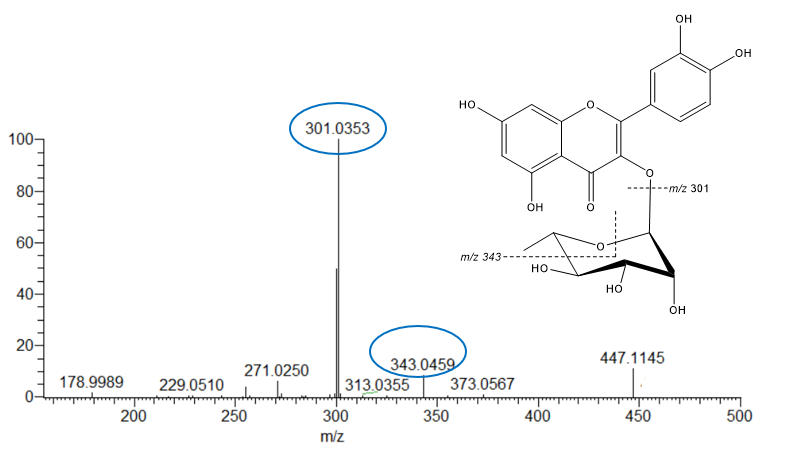
**

**Figure S2.** MS/MS fragmentation pattern of quercetin-*O*-deoxyhexoside

Supplement: S2 Fig — (DOCX) [file pone.0330595.s002.docx]

**
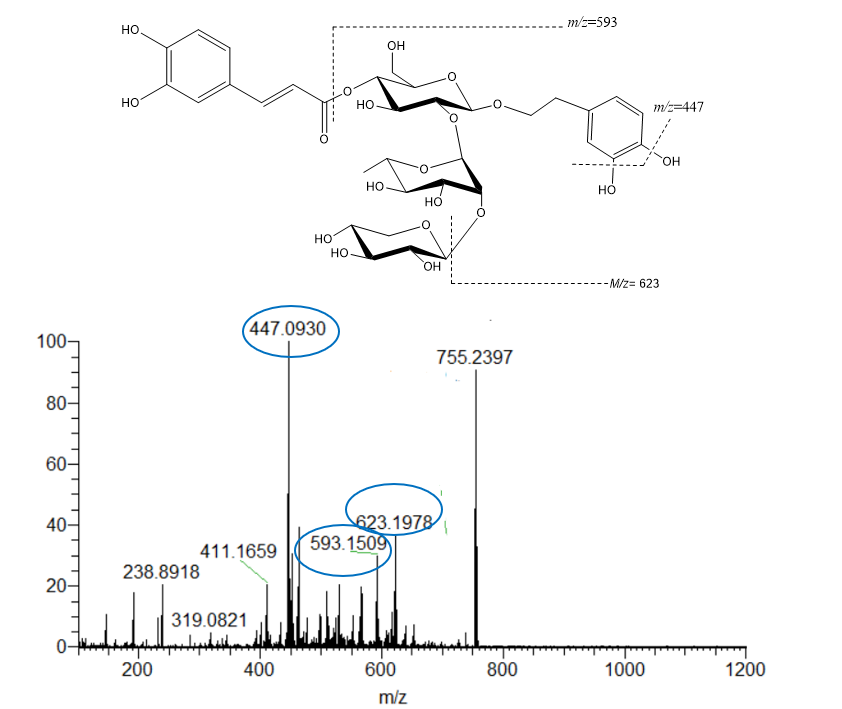
**

**Figure S3.** MS/MS fragmentation pattern of teucrioside

Supplement: S3 Fig — (DOCX) [file pone.0330595.s003.docx]
